# Supplementary material for: Self-supervised pseudo-colorizing of masked cells
Source: PLoS One. 2023 Aug 24;18(8):e0290561. doi: 10.1371/journal.pone.0290561 (PMC10449109; doi:10.1371/journal.pone.0290561)
Supplement: Attachment — Submitted filename: PLOS_ONE__rebuttal_letter.pdf [file pone.0290561.s001.pdf]

Rebuttal letter: Self-supervised pseudo-colorizing of masked cells

Response to reviewers

Reviewer 1: Thank you for your feedback.

Reviewer 2: Thank you for your feedback. Below are our responses to the points you raised during the review.
P: There are various datasets used and the reason behind using data augmentation is not clear.
R: We use only the simulated datasets for evaluation to use a perfect ground truth, since the other datasets do not contain perfect annotations, but mainly silver truth annotations generated from previous challenge submissions (see: `http://celltrackingchallenge.net/annotations`). However, we pre-compute the augmented samples to train all methods on the same data and make the comparison as fair as possible.
P: The computational complexity of the proposed method seems high.
R: We assume that you are referring to the higher computational complexity of our method compared to a vanilla masked autoencoder (MAE). MAE has a lower computational complexity since the vision transfomer (ViT) used as encoder is only applied to non-masked patches whereas the EfficientNet backbone in our model is applied to both masked and non-masked patches. Therefore, we additionally implemented a version of our CellCentroidFormer model with a ViT backbone to combine vanilla masked autoencoding with pseudo-colorizing and achieve the same computational efficiency. We added the results to Table 2, adjusted Fig 2, and published the code at: `https://github.com/roydenwa/pseudo-colorize-masked-cells`
